# Antagonist Effect of Triptolide on AKT Activation by Truncated Retinoid X Receptor-alpha

**DOI:** 10.1371/journal.pone.0035722

**Published:** 2012-04-24

**Authors:** Na Lu, Jinxing Liu, Jie Liu, Chunyun Zhang, Fuquan Jiang, Hua Wu, Liqun Chen, Wenjun Zeng, Xihua Cao, Tingdong Yan, Guanghui Wang, Hu Zhou, Bingzhen Lin, Xiaomei Yan, Xiao-kun Zhang, Jin-Zhang Zeng

**Affiliations:** 1 School of Pharmaceutical Sciences and Institute for Biomedical Research, Xiamen University, Xiamen, China; 2 Cancer Center, Sanford-Burnham Medical Research Institute, La Jolla, California, United States of America; 3 The Key Laboratory of Analytical Science, The Key Laboratory for Chemical Biology of Fujian Province, Department of Chemical Biology, College of Chemistry and Chemical Engineering, Xiamen University, Xiamen, China; Clermont Université, France

## Abstract

**Background:**

Retinoid X receptor-alpha (RXRα) is a key member of the nuclear receptor superfamily. We recently demonstrated that proteolytic cleavage of RXRα resulted in production of a truncated product, tRXRα, which promotes cancer cell survival by activating phosphatidylinositol-3-OH kinase (PI3K)/AKT pathway. However, how the tRXRα-mediated signaling pathway in cancer cells is regulated remains elusive.

**Methodology/Principal Findings:**

We screened a natural product library for tRXRα targeting leads and identified that triptolide, an active component isolated from traditional Chinese herb *Trypterygium wilfordii* Hook F, could modulate tRXRα-mediated cancer cell survival pathway *in vitro* and in animals. Our results reveal that triptolide strongly induces cancer cell apoptosis dependent on intracellular tRXRα expression levels, demonstrating that tRXRα serves as an important intracellular target of triptolide. We show that triptolide selectively induces tRXRα degradation and inhibits tRXRα-dependent AKT activity without affecting the full-length RXRα. Interestingly, such effects of triptolide are due to its activation of p38. Although triptolide also activates Erk1/2 and MAPK pathways, the effects of triptolide on tRXRα degradation and AKT activity are only reversed by p38 siRNA and p38 inhibitor. In addition, the p38 inhibitor potently inhibits tRXRα interaction with p85α leading to AKT inactivation. Our results demonstrate an interesting novel signaling interplay between p38 and AKT through tRXRα mediation. We finally show that targeting tRXRα by triptolide strongly activates TNFα death signaling and enhances the anticancer activity of other chemotherapies

**Conclusions/Significance:**

Our results identify triptolide as a new xenobiotic regulator of the tRXRα-dependent survival pathway and provide new insight into the mechanism by which triptolide acts to induce apoptosis of cancer cells. Triptolide represents one of the most promising therapeutic leads of natural products of traditional Chinese medicine with unfortunate side-effects. Our findings will offer new strategies to develop improved triptolide analogs for cancer therapy.

## Introduction

Retinoid X receptor-α (RXRα) is a unique member of the nuclear receptor superfamily [1], [2]. In addition to forming homodimer, RXRα also heterodimerizes with many other nuclear receptors such as retinoic acid receptor (RAR), peroxisome proliferator-activated receptor (PPAR), vitamin D_3_ receptor (VDR), thyroid hormone receptor (TR) and Nur77 orphan nuclear receptor [1], [2]. Thus, RXRα plays critical roles in regulating numerous cellular processes including cell growth, differentiation and apoptosis [1], [2], and the synthetic RXR ligand Targretin®/Bexarotene has been approved for treating cutaneous T-cell lymphoma [3]. Consistent to its profound effects, altered RXR expression and function are implicated in the pathogenesis of diseases and cancer. Diminished RXRα expression is associated with the development of certain malignancies, such as thyroid carcinoma [4], prostate cancer [5] and non-small-cell lung cancer [6]. RXRα ablation in adult tissues results in preneoplastic lesions in skin [7] and prostate [8]. In addition to reduced levels of RXRα protein, altered RXRα function by phosphorylation is associated with the development of human hepatocellular carcinoma [9], [10], [11] and colon cancer [12]. RXR binding to PML/RAR is essential for the development of acute promyelocytic leukemia [13], [14], further demonstrating the oncogenic potential of this protein when it acts inappropriately. Altered RXRα function can also be resulted from its proteolytic cleavage of the receptor protein, which is frequently observed in various human tumors [15], [16], [17], [18]. We recently reported our identification of an N-terminally truncated tRXRα protein in various cancer cells and in primary tumors but not in tumor surrounding or normal tissues [15]. Unlike full-length RXRα that resides in the nucleus, tRXRα is cytoplasmic and interacts with the p85α subunit of phosphatidylinositol-3-OH kinase (PI3K) to activate the PI3K/AKT pathway [15], a major survival pathway important for uncontrolled growth of tumor and its progression as well as drug resistance [19]. Thus, tRXRα acquires new function that is different from RXRα. Since tRXRα is often elevated in cancer cells, it is expected that targeting tRXRα represents a more effective and specific strategy for developing RXR-based anticancer drug. Thus, we show that non-steroidal anti-inflammatory drug sulindac and analogs bind to tRXRα and inhibit tRXRα-mediated PI3K/AKT activation *in vitro* and in animals [15].

Triptolide, a diterpene triepoxide, is a major active component of extracts derived from the medicinal plant *Tripterygium wilfordii* Hook F (TWHF) [20]. Triptolide has multiple pharmacological activities including anti-inflammatory, immune modulation, antiproliferative and proapoptotic activity [20], [21], [22]. It has been widely used to treat inflammatory diseases, autoimmune diseases, organ transplantation and even tumors [20], [23], [24], [25]. Despite its potent apoptotic effect, the underlying mechanisms by which triptolide induces apoptosis remain largely unclear. Triptolide has been found to activate p53 apoptotic pathways [26], [27], [28], to induce Bcl-2 cleavage and mitochondria dependent apoptosis [29], and to reduce the expression of cell cycle regulators [30] and survival genes such as cyclin D1 and Bcl-x [31]. In addition, Triptolide has been described to decrease the expression of heat shock proteins such as Hsp70, molecular chaperones associated with oncogenesis, by inactivation of heat shock transcription factor (HSF) [32], [33], and to inhibit transcription of numerous pro-inflammatory mediators [27], [34]. Interestingly, triptolide was shown to cooperate with tumor necrosis factor-α (TNFα) to induce apoptosis in tumor cells [35]. Here, we report that the apoptotic effect of triptolide is partially mediated by intracellular tRXRα expression in cancer cells. In addition, we show that triptolide selectively induces tRXRα degradation in cancer cells grown *in vitro* and in animals through its activation of p38 mitogen-activated protein kinase (p38 MAPK or p38). Furthermore, our results show that triptolide-induced p38 activation impairs tRXRα interaction with p85α, leading to inhibition of tRXRα-mediated AKT survival pathway. Our findings also demonstrate that triptolide enhances the apoptotic effect of chemotherapeutic agents and when used together with TNFα it strongly activates death receptor-mediated apoptotic pathway, showing a novel mechanism for shifting TNFα signaling from survival to death.

## Results

### Triptolide induces cancer cell apoptosis dependent on intracellular tRXRα expression

We recently reported that tRXRα, an N-terminally truncated form of RXRα, could strongly promote cancer cell growth through activation of PI3K/AKT pathway [15]. To further characterize the tRXRα-regulating pathway, we screened a natural product library of Chinese herbs for potential regulators. Our results show that triptolide strongly induces cancer cell apoptosis by regulating tRXRα expression and function.

**Figure 1:**
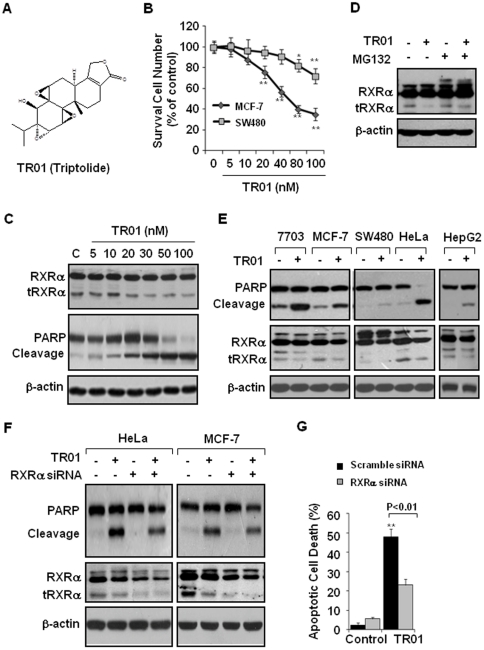
Triptolide induces cancer cell apoptosis dependent on intracellular tRXRα expression. (A) The chemical structure of triptolide. (B) Growth inhibitory effect. MCF-7 and SW480 cells were treated with various concentrations of triptolide as indicated. Cell viability was measured by the MTT colorimetric assay. *, *P*<0.05; **, *P*<0.01 (*vs* respective controls). (C) The effect of triptolide on tRXRα expression and PARP cleavage was examined in MCF-7 cells. The cells were treated with vehicle or increasing concentrations of triptolide for 9 h. (D) Triptolide induced proteasome-mediated tRXRα degradation. MCF-7 cells were treated with 50 nM triptolide with or without 10 µM MG132, a specific proteasome inhibitor. The impact of MG132 on tRXRα turnover was evaluated. (E) tRXRα expression was determined in various cancer cells as indicated. The apoptotic effects of triptolide in different cells were compared. The cells were treated with vehicle or 50 nM triptolide for 9 h. (F) HeLa and MCF-7 cells were transfected with scramble or RXRα siRNA and incubated with vehicle or 50 nM triptolide for 12 h. Triptolide-induced PARP cleavage was compared between control and RXRα siRNA transfections. (G) MCF-7 cells transfected with scramble or RXRα siRNA were treated with 50 nM triptolide for 12 h and subjected to DAPI staining. The apoptotic cells induced by triptolide were quantified and expressed as percentage of the counted cells.

We demonstrated that triptolide strongly induced growth inhibition in some cancer cell lines such as MCF-7 breast cancer cells, but with much less effect in others like SW480 colon cancer cells. Fig. 1B showed that MCF-7 cells significantly responded to triptolide at concentrations as low as 20 nM after 12 h treatment, while much higher concentrations (>80 nM) of triptolide were required to inhibit the growth of SW480 cells. Fig. 1C further showed that triptolide could dose-dependently induce apoptosis (PARP cleavage) in MCF-7 cells between 20 and 100 nM. Interestingly, triptolide-induced cancer cell apoptosis was closely associated with its decreasing tRXRα expression, while the levels of the full-length RXRα remained largely unaffected (Fig. 1C). The proteasome inhibitor MG132 was then used to evaluate the effect of triptolide on modulating tRXRα stability. Fig. 1D showed that triptolide-induced tRXRα reduction was greatly prevented by MG132, indicating that triptolide induces proteasome-mediated tRXRα degradation. To determine the role of tRXRα in regulating the apoptotic effect of triptolide, various cancer cell lines were recruited. Fig. 1E showed that tRXRα was highly expressed in QGY-7703 and HepG2 liver cancer cells, MCF-7 breast cancer cells, and HeLa cervical cancer cells, while level of tRXRα in SW480 colon cancer cells was hardly detectable. When the apoptotic effect of triptolide was examined, we found that the levels of tRXRα expression in these cancer cell lines were associated with their responses to the killing effect of triptolide. Triptolide-induced PARP cleavage was seen in the tRXRα-expressing cells but not in SW480 cells lacking tRXRα (Fig. 1E). In addition, triptolide showed no cytotoxic effect in non-cancerous HEK293T cells, which did not express tRXRα, even at high concentration of 100 nM (data not shown). To determine whether the intracellular tRXRα expression was essential for the death effect of triptolide, we transfected HeLa and MCF-7 cancer cells with RXRα siRNA, which effectively reduced the expression of both tRXRα and the full-length RXRα. Although the contribution of downregulation of the full-length RXRα to the apoptotic effect of triptolide was unknown, siRNA-mediated inhibition of tRXRα expression greatly impaired the effect of triptolide on inducing PARP cleavage in both HeLa and MCF-7 cancer cells (Fig. 1F). Consistently, when the apoptotic cells detected by DAPI staining were quantified [36], we found that treatment of MCF-7 cells with 50 nM for 12 h resulted in 48% cell death, while siRNA-mediated inhibition of tRXRα reduced this effect to about 23%. Our results clearly demonstrate that triptolide-induced cancer cell apoptosis is at least partially mediated by tRXRα.

### Triptolide suppresses tRXRα expression and tumor growth in animals

**Figure 2:**
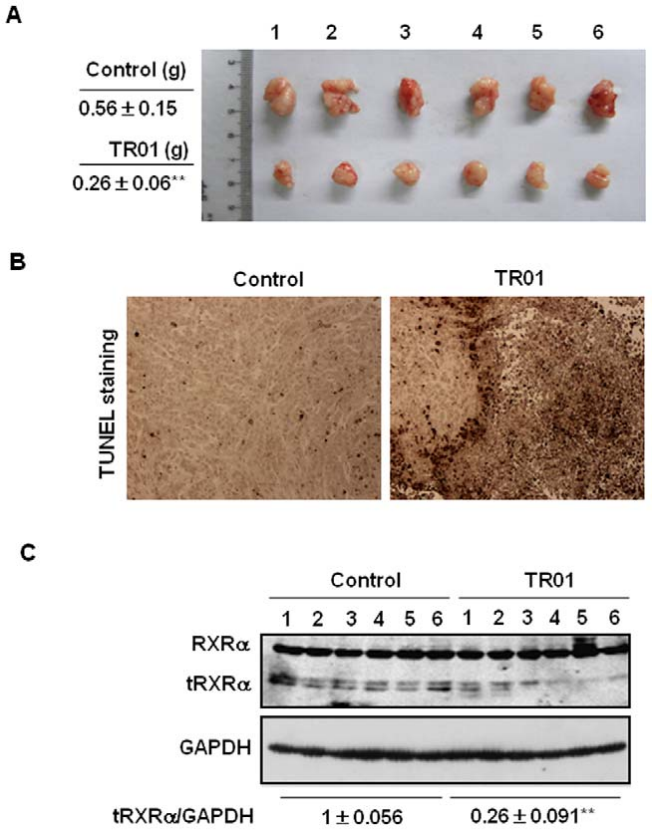
Triptolide induces tumor growth inhibition and tRXRα degradation *in vivo*. (A) Nude mice with HepG2 heptoma xenografts were intraperitoneally injected (*i.p.*) daily with saline or 0.2 mg/kg triptolide for 12 days. Tumor sizes and weights in control and triptolide-treated mice were compared. **, P<0.01 (vs control). (B) Tumor sections were stained for TUNEL by immunohistochemistry to show the apoptotic effect of triptolide. (C) The whole lysates prepared form HepG2 xenografts treated with triptolide or vehicle were subjected to Western blotting assays for detecting tRXRα expression. **, P<0.01 (*vs* control).

To further study the effect of triptolide on modulating tRXRα expression *in vivo*, mice with HepG2 tumor xenografts were treated with triptolide for 12 days. Administration of triptolide caused a 53.7% reduction of tumor volume (Fig. 2A) and extensive tumor cell apoptosis as indicated with brown TUNEL staining (Fig. 2B). Consistent with our *in vitro* observation, we showed that triptolide-induced tumor growth inhibition was closely associated with its inducing downregulation of tRXRα in the tumors (Fig. 2C). Our results demonstrate that tRXRα in cancer cells is a potential molecular target for the anticancer activity of triptolide *in vivo*.

### Triptolide induces tRXRα-mediated AKT inactivation and apoptosis

**Figure 3:**
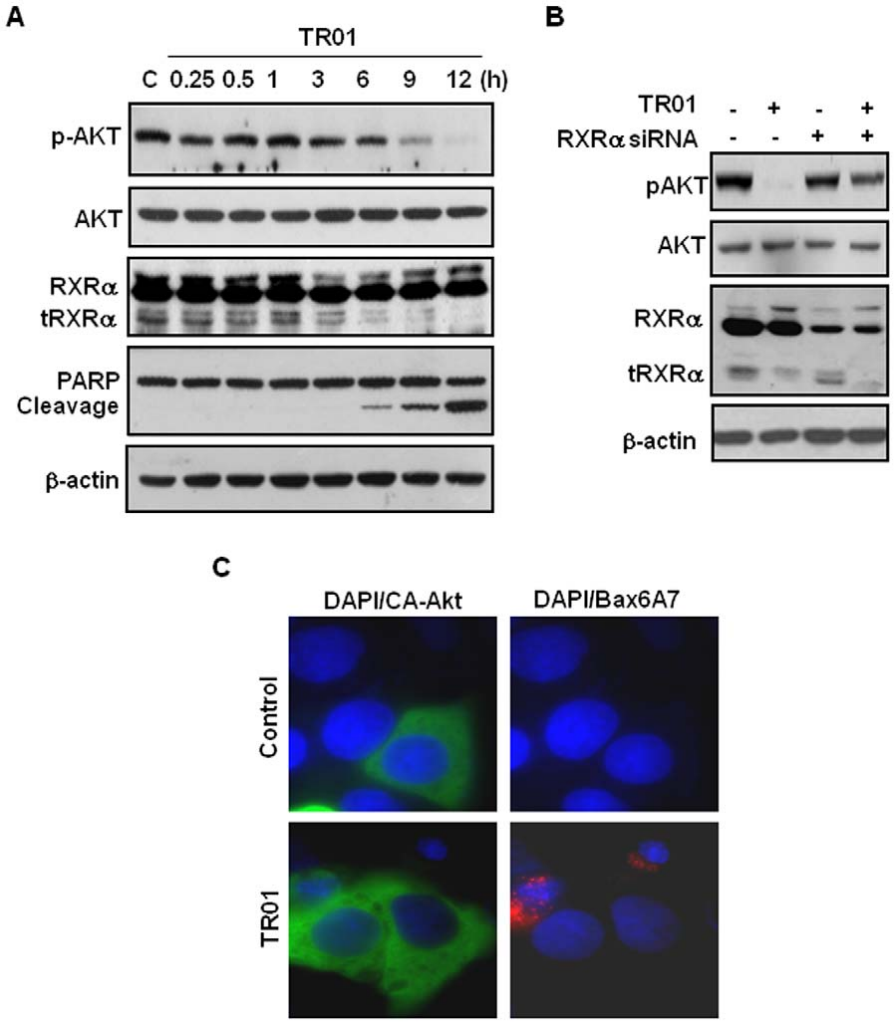
Triptolide inhibits tRXRα-dependent AKT activity and induces cancer cell apoptosis. (A) HepG2 cells were treated with vehicle or 50 nM triptolide for various time intervals as indicated. Time-dependent effects of triptolide on AKT activity, tRXRα degradation and PARP cleavage were examined. (B) Effect of RXRα siRNA. HepG2 cells transfected scramble or RXRα siRNA were treated with vehicle or 50 nM triptolide for 9 h. The effect of siRNA-mediated knocking down tRXRα expression on triptolide-inducing AKT dephosphorylation was studied. (C) Effect of CA-AKT. HepG2 cells were transiently transfected with active form of AKT expression vector (GFP-CA-AKT) and treated with 80 nM triptolide for 12 h. Apoptotic cells (condensed and fragmentated) induced by triptolide were recognized by DAPI staining, while Bax activation was detected by conformation-sensitive Bax/6A7 antibody.

We previously reported that the oncogenic activity of tRXRα was due to its activation of the AKT survival pathway [15]. We then investigated whether triptolide could inhibit tRXRα-dependent AKT activation. Indeed, treatment of HepG2 liver cancer cells with triptolide resulted in a sustained inhibition of AKT phosphorylation from 6 h after treatment, which was closely associated with its inducing tRXRα degradation (Fig. 3A). To study the role of tRXRα in triptolide inactivation of AKT, HepG2 cells were transfected with RXRα siRNA. Fig. 3B showed that treatment of HepG2 cells with 50 nM triptolide for 9 h completely inhibited AKT phosphorylation, while knocking down tRXRα expression by siRNA greatly impaired triptolide on inducing AKT dephosphorylation. These studies demonstrate that tRXRα expression is required for triptolide to inactivate AKT. Our results showed that triptolide-induced tRXRα degradation and AKT inactivation were closely associated with its apoptotic effect (Fig. 3A). To determine whether triptolide inhibition of AKT activity was responsible for its induction of apoptosis in cancer cells, HepG2 cells transfected with a constitutive-active form of AKT (CA-AKT) were treated with triptolide and the apoptotic effect of triptolide was assayed. Fig. 3C showed that triptolide-induced nuclear condensation and fragmentation frequently found in untransfected cells were inhibited in CA-AKT transfected cells. Consistently, triptolide-induced Bax activation as revealed by immunostaining of cells with conformation-sensitive Bax/6A7 antibody [37] was also inhibited by CA-AKT expression (Fig. 3C). Triptolide-induced AKT inactivation and apoptosis were also reproducible in several other cancer cell lines including MCF-7 breast cancer cells and A549 lung cancer cells (data not shown).

### Triptolide inhibits TNFα-induced AKT activation

**Figure 4:**
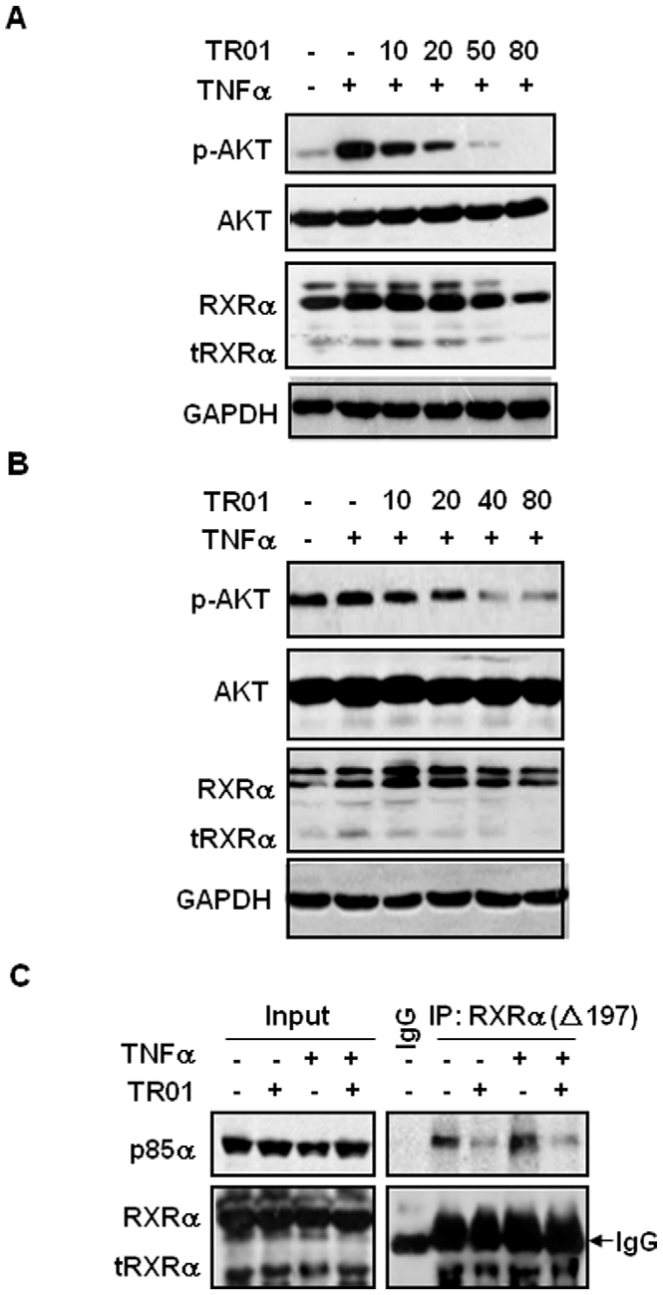
Triptolide inhibits TNFα-induced AKT activation. (A, B) The effect of triptolide on TNFα-induced AKT phosphorylation was determined in MCF-7 cells (A) and A549 cells (B). Cells were treated with vehicle or 10 ng/ml TNFα in the absence or presence of increasing concentrations of triptolide for 12 h. (C) Co-immunoprecipitate assays were carried out in MCF-7 cells to determine tRXRα interaction with p85α. The cells were treated with vehicle or 50 nM triptolide in the absence or presence of 10 ng/ml TNFα for 6 h. Cell lysates were immunoprecipitated with ΔN197 anti-RXRα antibody. The coimmunoprecipitates were then subjected to Western blotting analysis for tRXRα expression and its co-precipitated p85α by ▵N197 anti-RXRα and anti-p85α antibodies respectively.

TNFα is known to induce both apoptotic and survival pathways [38]. We previously showed that one of the survival signaling pathways of TNFα was mediated by tRXRα-dependent AKT activation [15]. Interestingly, triptolide was shown to sensitize tumor cells to TNFα-induced apoptosis [35]. To investigate whether triptolide could inhibit TNFα-induced AKT activation, MCF-7 cells were treated with vehicle or 10 nM TNFα in the presence or absence of triptolide. In agreement with previous results [15], immunoblotting assays showed that TNFα strongly induced AKT activation in these cells, which was inhibited by triptolide in a dose-dependent manner (Fig. 4A). Consistently, the inhibitory effect of triptolide on AKT activation was associated with decrease of tRXRα expression (Fig. 4A). Such effects of triptolide were also observed in A549 lung cancer cells (Fig. 4B). The role of tRXRα in triptolide inhibition of TNF-induced AKT activation was then determined by studying the effect of triptolide on TNFα-induced tRXRα interaction with p85α, an event that leads to activation of the PI3K/AKT pathway [15]. Co-immunoprecipitation assays showed that endogenous p85α in MCF-7 cells could be immunoprecipitated together with tRXRα by ▵N197 anti-RXRα antibody but not by IgG (Fig. 4C). Interaction of p85α with tRXRα was enhanced by TNFα. When cells were treated with triptolide, both basal and TNFα-induced tRXRα interaction with p85α was strongly inhibited (Fig. 4C), demonstrating that triptolide-induced inhibition of AKT activation is due to its inhibition of tRXRα interaction with p85α.

### Triptolide induces mitochondrial-mediated caspase 9-dependent apoptosis and activates caspase 8-dependent apoptotic pathways by TNFα

**Figure 5:**
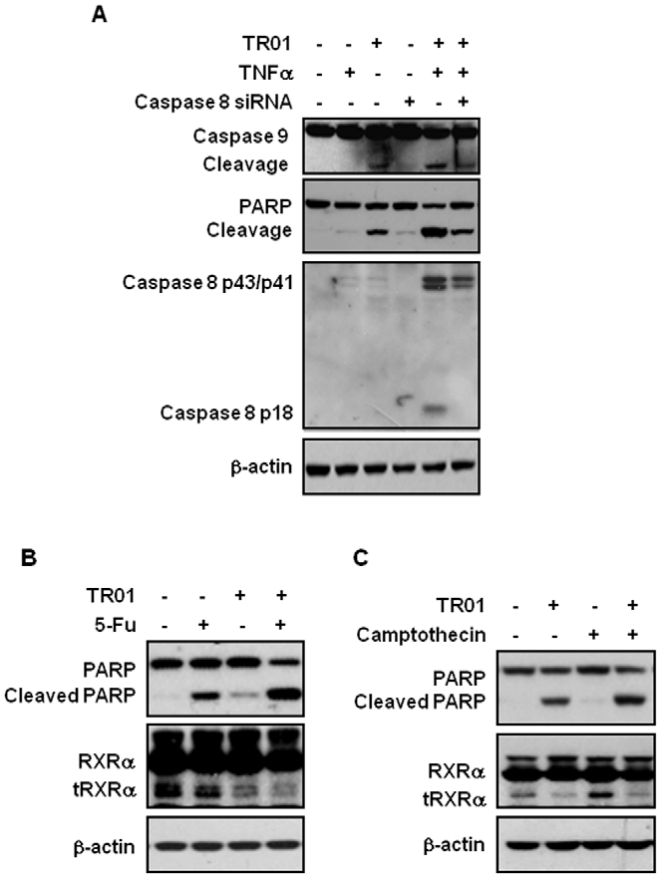
Triptolide enhances the apoptotic effect of TNFα and other chemotherapies. (A) MCF-7 cells were transfected with caspase 8 siRNA to evaluate whether triptolide could activate TNFα-dependent death effect. Untransfected and transfected cells were treated with vehicle or 50 nM triptolide with or without 10 ng/ml TNFα for 12 h. Expression and cleavages of caspase 8, 9 and PARP were analyzed. (B, C) Triptolide-enhanced the apoptotic effect of 5-Fu and camptothecin was examined in HepG2 (B) and MCF-7 cells (C) respectively. Cells were treated with 50 nM triptolide alone or in combination with 10 µM 5-Fu or 10 µM camptothecin for 9 h.

To further determine the apoptotic effect of triptolide, we examined caspase 8, 9 and PARP cleavages in MCF-7 cells. Fig. 5A showed that triptolide strongly increased caspase 9 and PARP cleavages, while it failed to activate caspase 8, indicating that triptolide can alone induce mitochondrial-activated apoptosis. Consistently, triptolide has been shown to be inefficient for apoptosis induction in caspase 9 knock-out cells but remains sensitive in caspase 8 deficient cells [39]. TNFα is known to induce not only cell survival and proliferation through its activation of PI3K/AKT and IKK/NF-κB pathways [15], [40] but also cell death through its activation of death receptor-dependent apoptotic pathway [38]. We then determined whether the ability of triptolide to inhibit TNFα activation of AKT could result in TNFα activation of caspase 8-dependent apoptotic pathway [15]. Fig. 5A showed that TNFα alone could not induce PARP cleavage and had no appreciable effect on caspases 8 and 9, consistent with the notion that the apoptotic pathway of TNFα is usually inactivated in cancer cells [40]. However, when MCF-7 cells were co-treated with triptolide and TNFα, we observed proteolytical cleavage of caspase 8 into p43, p41, and p18 active forms, suggesting that triptolide is able to activate TNFα-dependent apoptosis pathway. Induction of TNFα-dependent apoptosis by triptolide contributed to overall death effect of triptolide as TNFα and triptolide combination resulted in synergistic apoptotic effect. This was also illustrated by our observation that knocking down caspase 8 expression by siRNA transfection impaired the synergistic effect of triptolide and TNFα. Consistently, triptolide was described to sensitize lung cancer cells to TNF-induced apoptosis through TNF-related apoptosis-inducing ligand (TRAIL) [41]. Thus, these results demonstrate that the death effect of TNFα can be induced by triptolide.

We then showed that targeting tRXRα by triptolide could also significantly enhance the apoptotic responses of other chemotherapies such as 5-Fu in HepG2 liver cancer cells (Fig. 5B) and camptothecin in MCF-7 breast cancer cells (Fig. 5C). Both 5-Fu and camptothecin could not alter the basal and triptolide-reducing tRXRα expression.

### p38 is involved in triptolide inhibition of tRXRα-dependent AKT activation

**Figure 6:**
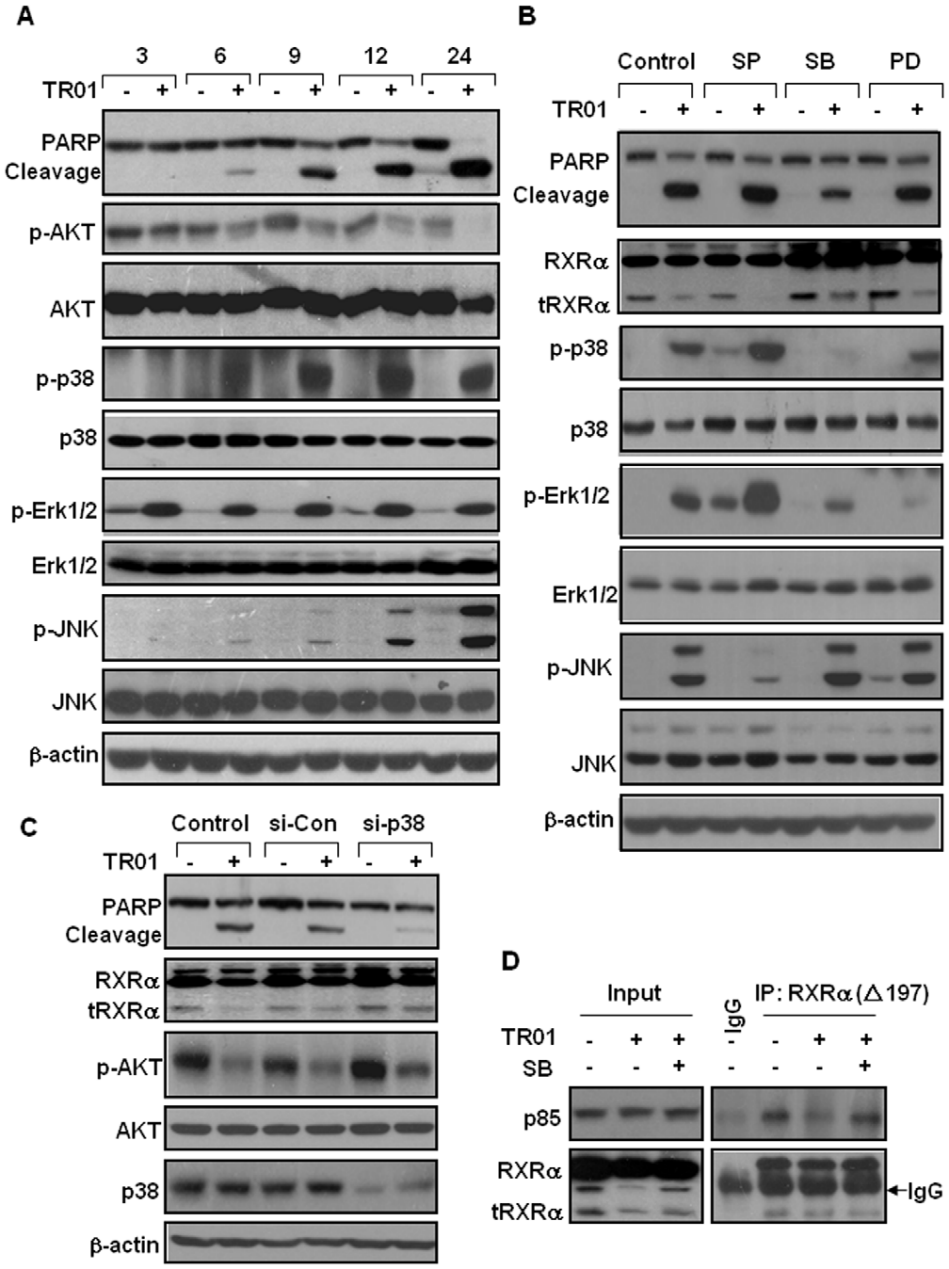
Triptolide induces tRXRα degradation and AKT inactivation through activation of p38. (A) Triptolide induced activation of several MAPK pathways. HepG2 cells were treated with vehicle or 50 nM triptolide for various time intervals as indicated. Triptolide-induced time-dependent phosphorylation of p38, JNK and Erk1/2 was compared to its effect on decreasing AKT phosphorylation and PARP cleavage. (B) HepG2 cells were treated with 50 nM triptolide for 9 h with or without p38 inhibitor SB203580 (10 µM), JNK inhibitor SP600125 (10 µM) or Erk1/2 MAPK inhibitor PD98059 (10 µM). The impact of inhibition of the individual pathways on tRXRα degradation and PARP cleavage was determined. (C) HepG2 cells transfected with scramble or p38 siRNAs were treated with vehicle or 50 nM triptolide for 9 h. The effect of siRNA-mediated p38 inhibition on triptolide inactivation of AKT and tRXRα degradation was assayed. (D) HepG2 cells were treated with vehicle or 50 nM triptolide for 9 h in the presence or absence of SB203580. The lysates were immunoprecipitated with ΔN197 anti-RXRα antibody and analyzed for its co-immunoprecipitated with p85α.

One way that triptolide-induced tRXRα degradation is through its binding to the receptor protein. However, our classical ligand competition binding assays failed to detect any binding of triptolide to purified RXRα protein (data not shown). We then reasoned that triptolide might act indirectly to modulate the stability of tRXRα protein and its association with AKT activation. Our investigation of the effect of triptolide on MAPK signal transduction pathways in HepG2 cells revealed that triptolide could strongly activate Erk1/2, p38, and JNK1/2 (Fig. 6A). The time course assays showed that triptolide activation of p38 was in parallel with its inhibition of AKT activity and PARP cleavage. To study the causal role of p38 in triptolide modulation of tRXRα-dependent AKT activity, HepG2 cells were treated with 50 nM triptolide in the presence or absence of p38 inhibitor SB203580, while JNK inhibitor SP600125 and ERK1/2 inhibitor PD98059 were similarly used for comparison. Fig. 6B showed that triptolide-induced tRXRα degradation and PARP cleavage were significantly inhibited by SB203580 but not by SP600125 and PD98059, demonstrating that p38 is involved in regulating tRXRα turnover and apoptosis by triptolide. Consistently, knocking-down p38 by siRNA transfection reduced the inhibitory effects of triptolide on tRXRα stability, AKT activation and PARP cleavage (Fig. 6C). Furthermore, we observed that triptolide failed to inhibit the interaction of tRXRα with p85α in the presence of SB203580 (Fig. 6D). Together, our results demonstrate that p38 activation by triptolide is essential for its inactivation of tRXRα-dependent AKT pathway and its apoptotic effect.

## Discussion

We recently demonstrated that truncated RXRα, tRXRα, resulted from limited proteolytic cleavage of RXRα in several human tumors as well as in a number of cancer cell lines, confers tumor growth advantage due to its activation of PI3K/AKT survival signaling [15]. Here, we report that triptolide isolated from Chinese medicinal herb *Trypterygium wilfordii* Hook F is a new regulator of tRXRα-mediated signaling pathway.

We show that the levels of tRXRα in cancer cells determine their apoptotic responses to triptolide (Fig. 1E and F). Triptolide strongly induces PARP cleavage in tRXRα-expressing cells including QGY-7703 and HepG2 liver cancer cells, MCF-7 breast cancer cells, and HeLa cervical cancer cells, while it has little effect in SW480 colon cancer cells and HEK293T non-cancerous cells that express trace amount of tRXRα (Fig. 1E and data not shown). Knocking down tRXRα expression by siRNA greatly impairs the death effect of triptolide in cancer cells (Fig. 1F and G). These findings suggest that tRXRα protein serves as one of the important targets of triptolide action.

Our results reveal that the apoptotic effect of triptolide in cancer cells is closely associated with its inducing tRXRα degradation *in vitro* (Fig. 1) and *in vivo* (Fig. 2). Targeting tRXRα for degradation by triptolide results in reduction of AKT activity (Fig. 3A). In addition, triptolide strongly inhibits basal and TNFα-induced AKT activity through disrupting the interaction between tRXRα and p85α (Fig. 4C). Triptolide inactivation of tRXRα-dependent AKT is critical for its apoptotic induction, which is illustrated in Fig. 3D showing that triptolide-induced cancer cell apoptosis and activation of pro-apoptotic molecule Bax are inhibited by transfection of constitutive-active AKT.

Interestingly, the effect of triptolide on tRXRα stability and AKT inactivation is due to its activation of p38 rather than through directly binding to tRXRα. Our time-course assays show that AKT inactivation by triptolide is closely correlated with its activation of p38 (Fig. 6A). Inhibition of p38 by p38 siRNA transfection or treatment with the p38 inhibitor SB203580 diminishes the effects of triptolide on inducing tRXRα degradation and inhibiting tRXRα-mediated AKT activation (Fig. 6B and C). In addition, triptolide-induced inhibition of tRXRα interaction with p85α is blocked by SB203580 (Fig. 6D). Although triptolide also strongly activates JNK and Erk1/2, inhibition of both kinases does not exert significant effect on tRXRα stability and cancer cell apoptosis (Fig. 6B and C). p38 is typically a stress-activated kinase that promotes inflammation, and is frequently deregulated in cancers, in which it exerts both tumor suppressive and promoting effects [42], [43]. Interestingly, the apoptotic effect of p38 is often antagonized by AKT, and it is suggested that the cell fate is often determined by the balance of AKT and p38 activities [42]. Our findings reveal that activation of p38 by triptolide results in suppression of AKT activity and cancer cell apoptosis through mediation of tRXRα, a novel mechanism for balancing the activities of p38 and AKT.

We also demonstrate that targeting tRXRα by triptolide strongly activates TNFα death signaling. TNFα is a multifunctional cytokine that plays roles in diverse cellular events such as cell survival and death [38], [40]. Although TNFα can be a potent death-inducing factor of cancer cells, its killing effects are often antagonized by its survival function that is mainly mediated by activation of the NF-κB and PI3K/AKT pathways [40]. Triptolide was shown previously to sensitize cancer cells to TNFα-induced apoptosis [35]. We show here that triptolide activates TNFα-dependent caspase 8-mediated apoptosis through targeting tRXRα oncogenic protein (Fig. 4 and 5). The combination of TNFα and triptolide results in stimulation of both extrinsic and intrinsic apoptotic pathways, thus contributing to greater apoptotic effect in cancer cells. The tRXRα-dependent apoptotic effect of triptolide also significantly promotes the anticancer activity of other chemotherapies such as 5-Fu, which is shown to use Fas/FasL pathway [44], [45] and requires thymine DNA glycosylase for its anticancer activity [46], and camptothecin, which potently disrupts DNA processing by inhibition of topoisomerase I [47].

In summary, we demonstrate that triptolide serves as an important regulator of tRXRα-mediated cancer cell survival pathway by targeting the tumor-specific tRXRα protein through an interesting novel signaling interplay between p38 and AKT. Triptolide and analogs have recently been received wide attention as these chemicals show promising anticancer activity *in vitro* and *in vivo* [20]. However, their significant side effects still limit these compounds for clinical use. Thus, our findings provide useful molecular basis for developing improved triptolide-based cancer therapeutics.

## Materials and Methods

### Reagents

Lipofectamin 2000 was purchased from Invitrogen. Goat anti-rabbit and anti-mouse secondary antibodies conjugated to horseradish peroxidase and enhanced chemilumienescence(ECL) reagents were from Thermo. Polyclonal antibodies against RXRα (ΔN197), AKT1/2/3 (H-136), Cyclin D1 (H-295), and monoclonal antibodies against Bax (6A7), GFP (B-2), c-Myc (9E10), GFP- and FITC-labeled anti-rabbit IgG were from Santa Cruz Biotechnology. Polyclonal antibodies against p38 and PARP, and monoclonal antibodies against p-AKT (D9E), cleaved caspase 8 (Asp391), p-p38 (3D7), Erk1/2 (C-16), p-Erk1/2 (D13.14.4E), p-JNK (81E11), and JNK (2C6) were from Cell Signaling Technology. Polyclonal p85α antibody was from Millipore and anti-mouse IgG conjugated with Cy3 from Chemicon. Monoclonal antibodies against glyceraldehyde-3-phosphatedehydro-genase (GAPDH) and β-actin, and chemicals including tripotide, camptothecin, 5-fluorouracil (5-Fu), MG132, SP600125, SB203580, TNFα, epidermal growth factor (EGF) were from Sigma. Protein A beads were from GE Healthcare and polyvinylidene difluoride (PVDF) membrane from Millipore. TUNEL kit was from Roche. The cocktail of proteinase inhibitors were from Amersham.

### Cell lines

HepG2 (ATCC HB-8065), MCF-7 (ATCC HTB-22), HeLa (ATCC CCL-2), A549 (ATCC CCL185), SW480 (ATCC CCL-228), HEK293T (ATCC CRL-11268) and QGY-7703 (from Institute of Biochemistry and Cell Biology, SIBS, CAS) [48].

### siRNAs

Several siRNA oligos were synthesized (Ribobio Co, Guangzhou, China). siRNA sequence for p38 used in this study is: 5′-GGAATTCAATGATGTGTAT-3′, while ERK1/2 siRNAs include a mixture of the following sequences: 5′-CGTCTAATATATAAATATA-3′, 5′-CCCTGACCCGTCTAATATA-3′, 5′-CACTTGTCAAGAAGCGTTA-3′, 5′-CATGGTAGTCACTAACATA-3′. The sequences for RXRα siRNA (M-003443-02), caspase 8 siRNA (J-003466-14), and control siRNA (D-001206-09-05) were described previously [15].

### Cell Culture and Transfection

Cells were cultured in DMEM containing 10% fetal bovine serum (FBS) in a humidified atmosphere containing 5% CO_2_ at 37°C. Subconfluent cells with exponential growth were used throughout the experiments. Transfections were carried out by using Lipofectamine 2000 according to the instructions of the manufacturer.

### MTT assays

Confluent cells cultured in 96-well dishes were treated with various concentrations of triptolide for 12 h. The cells were then incubated with 2 mg/ml MTT for 1 h at 37°C and dissolved with 1 ml of dimethyl sulfoxide. Cell viability was measured based on MTT dye conversion at 570 nm.

### Apoptosis assays

MCF-7 cells grown on 35-mm culture dishes were transfected with RXRα siRNA or scramble siRNA. After 48 h of transfection, cells were incubated with vehicle or with 50 nM triptolide in serum-free medium for 12 h. Detached and attached cells were collected for DAPI staining. Apoptotic cells were counted as previously described [36].

### HepG2 Xenografts

Nude mice (BALB/c, SPF grade, 16–18 g, 4–5-week old) were housed at 28°C in a laminar flow under sterilized conditions. Mice were subcutaneously implanted with 200 µl HepG2 cell suspension (5×10^6^ cells/per mouse). Mice were intraperitoneally injected with 0.2 mg/kg triptolide or vehicle daily after 7 days of transplantation. Food consumption, body weight and tumor sizes of mice were measured every other day. Mice were scarified after 12-day drug treatment and the tumors removed for various assessments. The study was approved by the ethics committee of Xiamen University.

### Immunohistochemistry

Tumor sections of HepG2 xenografts were stained with TUNEL for assessing spontaneous apoptosis according to the manufacturer's instructions (*In situ* Cell Death Detection Kit; Roche). The effect of AKT on modulating the apoptotic effect of triptolide was determined in HepG2 liver cancer cells transfected with GFP-CA-AKT. Cells were mounted on glass slides and treated with vehicle or 80 nM triptolide for 12 h. The slides were incubated with anti-Bax (6A7, 1∶100) antibody and detected by anti-mouse IgG conjugated with Cy3 (1∶100). Cells were co-stained with 4′6′-diamidino-2-phenylindole (DAPI) to visualize nuclei. The images were taken under a fluorescent microscope (Carl Zeiss).

### Co-immunoprecipitations

Cells were lysed in buffer containing 50 mM Hepes-NaOH (pH 7.5), 2.5 mM EDTA, 100 mM NaCl, 0.5% NP40, and 10% glycerol, with 1 mM DTT and proteinase inhibitor cocktail. Whole cell lysates were subjected to immunoprecipitation with anti-RXRα (ΔN197) as described [15].

### Western Blotting

A cocktail of proteinase inhibitors were included in all protein purification. Equal proteins were electrophoresed on an 8% SDS-PAGE gel and transferred onto PVDF membranes. The membranes were incubated with primary and secondary antibodies as indicated and detected using ECL system. The antibodies used in these assays included: RXRα (ΔN197; 1∶1000), PARP (1∶1000), β-actin (1∶10000), GAPDH (1∶1000), Myc (1∶2000), GFP (1∶1000), AKT (1∶1000), p-AKT (1∶500), p85α (1∶1000), cyclin D1 (1∶1000), caspase 8 (1∶500), p38 (1∶1000), p-p38 (1∶500), Erk (1∶1000), p-Erk1/2 (1∶2000), JNK (1∶1000) and p-JNK (1∶1000). All data provided in the results are representative of at least three experiments.

### Isolation and purification of triptolide

Triptolide was isolated from the roots of Chinese herb *Tripterygium wilfordii* Hook F (TWHF) and its structure was identified using a combination of chromatographic techniques and nuclear magnetic resonance analysis. The purity of triptolide used in this study was more than 98%. Triptolide was dissolved in DMSO and stored as a stock at 10^−2^ M at −80°C. The working concentrations of triptolide and the vehicle controls used in this study contained 0.1% DMSO, a concentration which did not alter cell function.

### Statistical Analysis

Data were expressed as mean ± SD from three or more experiments. Statistical analysis was performed using Student's t-test. Differences were considered statistically significant with p<0.05.
